# A lethal course of hypertrophic cardiomyopathy in Noonan syndrome due to a novel germline mutation in the *KRAS* gene: case study

**DOI:** 10.3325/cmj.2013.54.574

**Published:** 2013-12

**Authors:** Gregor Nosan, Sara Bertok, Samo Vesel, Helger G. Yntema, Darja Paro-Panjan

**Affiliations:** 1 Department of Neonatology, Division of Pediatrics, University Medical Centre Ljubljana, Ljubljana, Slovenia; 2 Department of Endocrinology, Diabetes and Metabolic Diseases, Division of Pediatrics, University Medical Centre Ljubljana, Ljubljana, Slovenia; 3 Cardiology Unit, Division of Pediatrics, University Medical Centre Ljubljana, Ljubljana, Slovenia; 4 Department of Human Genetics, Radboud University Medical Centre, Nijmegen, the Netherlands

## Abstract

Noonan syndrome is a relatively common and heterogeneous genetic disorder, including congenital heart defect in more than half of the cases. If the defect is not large, life expectancy is normal. Here we report on a case of an infant with Noonan syndrome and rapidly progressive hypertrophic cardiomyopathy with lethal outcome, in whom we identified a novel mutation in the *KRAS* gene. This heterozygous unclassified missense variant in exon 3: c.179G>T (p.Gly60Val) might be associated with a lethal form of Noonan syndrome. The malignant clinical course of the disease and the lethal outcome in an infant only a few months old might be connected to RAS-mitogen-activated protein kinase pathway hyperactivation, consequently promoting cell growth and proliferation, leading to rapidly progressive hypertrophic cardiomyopathy. Further biochemical and functional studies are needed to confirm this hypothesis.

Noonan syndrome (NS; *http://www.omim.org/entry/163950?search=163950&highlight=163950*) is a relatively common genetic disorder with an incidence of 1 per 1000-2500 live births (1). Clinically it is a very heterogeneous disorder, predominantly characterized by dysmorphic facial features, congenital heart defect (CHD), post-natal short stature, webbed neck, chest deformity, cryptorchidism in men, lymphatic dysplasia, variable bleeding disorders, and intellectual disability. CHD is present in 50 to 80% of affected individuals and it is also very heterogeneous (2). Most commonly found are pulmonary valve stenosis with or without dysplastic pulmonary valve and hypertrophic cardiomyopathy. Providing the CHD is not large, life expectancy is in the normal range (3). NS and CHD are regularly connected with germline *KRAS* mutations. We describe a patient with NS and rapidly progressive hypertrophic cardiomyopathy with lethal outcome, in whom we identified a novel mutation in the *KRAS* gene.

## Case report

**Figure 1:**
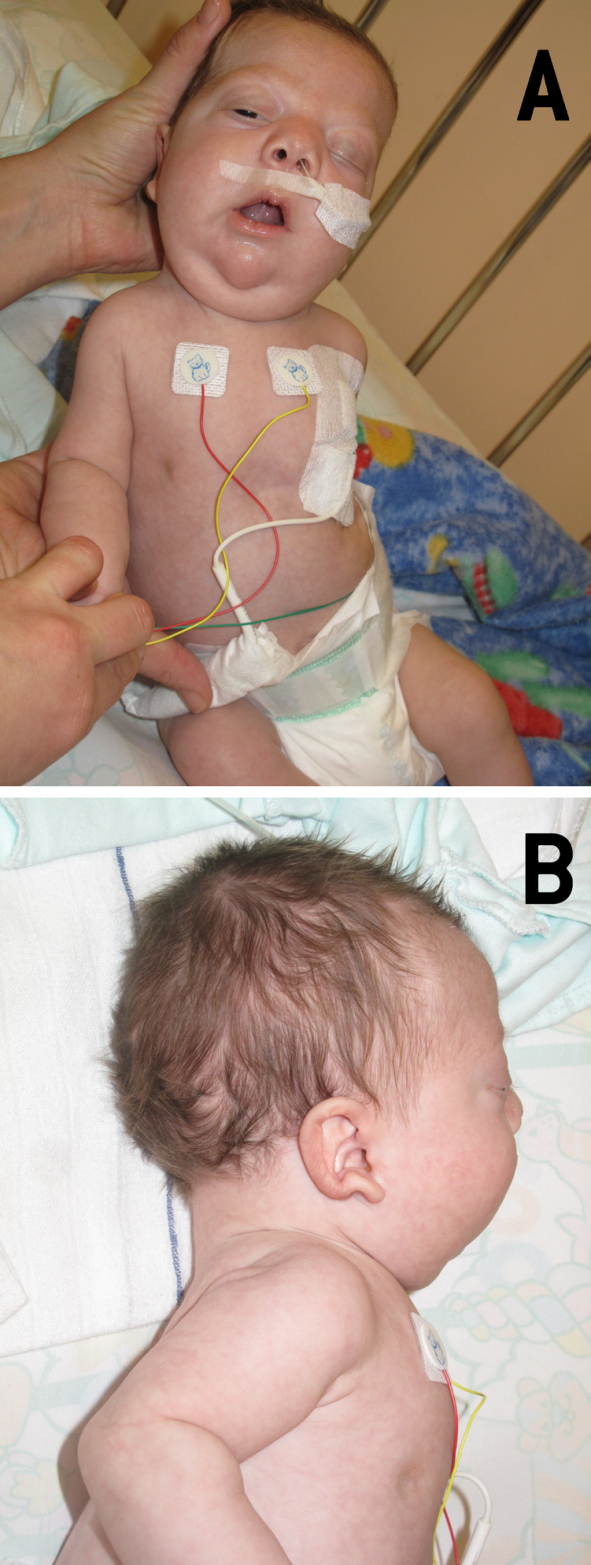
Dysmorphic features of the patient with Noonan syndrome at the age of 3 months. (**A**) antimongoloid palpebral slant, left side ptosis, (**B**) low set, posteriorly rotated, and dysmorphic ear. Patient’s images are published with parents’ consent.

The patient was born to healthy Caucasian non-consanguineous parents who already had a healthy 4-year old daughter. It was the mother’s second pregnancy, which was complicated by polyhydramnios and increased fetal nuchal translucency thickness. Chorionic villus biopsy was performed and revealed normal male karyotype. Several fetal morphology ultrasounds and two fetal echocardiography examinations revealed no abnormalities. The labor started spontaneously after 33 weeks of pregnancy, the delivery was vaginal, and the amniotic fluid was meconium stained. Birth weight was 2780 g (95th percentile), length 48 cm (90th percentile), head circumference 33.5 cm (90th percentile), and Apgar score 5/7/8. After birth, diffuse lymphedema of the body and several dysmorphic features (Figure 1) were identified: frontal bossing, antimongoloid palpebral slant, exophthalmos, left sided ptosis, wide nasal tip, low set, posteriorly rotated, and dysmorphic ears, thickened philtrum, tented upper lip, micrognathia, high arched palate, large neck skin fold, posterior low set hair line, and right scrotal hernia. Echocardiography was performed due to heart murmur and patent ductus arteriosus and mild coarctation of aorta were found; during short follow-up the ductus closed spontaneously and the coarctation remained unchanged. At the age of 2 weeks, echocardiography was repeated and revealed hypertrophy of both ventricles, and thickened and dysplastic atrioventricular and semilunar valves. In addition, there was also a small atrial septal defect with left-to-right shunt and mild aortic coarctation. Ultrasound of the abdomen revealed mild right-sided hydronephrosis and ectopic dilated right urether. Ultrasound of the head showed no structural anomalies. Ophthalmologic evaluation was unremarkable. The clinical picture highly indicated NS.

DNA sequence analysis from peripheral blood leukocytes was performed after the informed consent from parents had been received. Sanger sequencing of the entire coding regions of the genes involved in NS was performed at the Department of Human Genetics, Radboud University Medical Centre, Nijmegen. All coding exons and flanking intronic sequences of genes involved in NS were polymerase chain reaction (PCR)-amplified, and direct sequencing was performed on an ABI 3730 automated DNA sequencer (Applied Biosystems, Foster City CA, USA). 100 ng DNA was amplified with Amplitaq Gold 360 Master Mix (Life Technologies: Bleiswijk, the Netherlands) in the presence of 5 pmol of each primer, forward (f) and reverse (r). Cycling conditions were 30 seconds at 95°C, 30 seconds at 60°C, and 1 minute at 72°C (35 cycles). For the *KRAS* gene the following primer sequencing (5′-3′) were used: exon 2: gtctgcagtcaactggaatt (f) and ccaaggaaagtaaagttccc (r), exon 3: gcatcttttcaggtgcttag (f) and acagggatattacctacctc (r), exon 4: ggtgtagtggaaactaggaa (f) and accaaagccaaaagcagtac (r), exon 5: gaacaaaccaggattctagc (f) and gtagttctaaagtggttgcc (r), exon 6: cagttgcctgaagagaaaca (f) and ccaaaactctgggaatactg (r).

**Figure 2:**
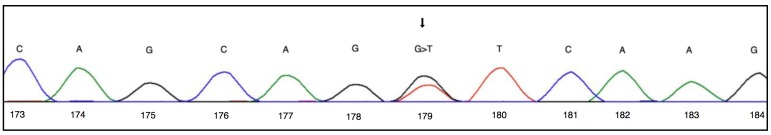
A novel mutation in exon 3 of the *KRAS* gene. The cytogenetic location of the *KRAS* gene is on the short (p) arm of the chromosome 12 at the position 12.1. *KRAS* gene comprises four exons spanning 45 kb, more precisely from base pair 25.358.179 to base pair 25.403.869 on the chromosome 12. DNA sequencing of the exone 3 and a heterozygous missense variant at the codone 179, conversing guanine to thymine (c.179G>T). The G>T conversion turns 179th triplet coding for glycine into valine (p.Gly60Val).

A heterozygous unclassified variant in the *KRAS* gene was detected and NS was confirmed. It was a missense variant in exon 3: c.179G>T (p.Gly60Val) (Figure 2). Furthermore, genotyping of parental DNAs demonstrated that the mutation in the child occurred de novo.

Patient’s clinical state deteriorated at the age of 28 days, when bacterial pneumonia developed; due to acute respiratory failure he needed artificial ventilatory support for almost one month and for the same reason again at the age of 3 months. Cardiac function deteriorated significantly due to rapidly progressive hypertrophic cardiomyopathy and apart from ventilatory support he also received treatment with propranolol and high doses of thiazide diuretics. As the heart disease was progressing rapidly and the diagnosis of NS was genetically confirmed, the parents agreed to withhold any further intensive treatment. He died at the age of four months due to a cardiorespiratory failure. The post-mortem autopsy was not performed.

## Discussion

There are three clinically important *RAS* genes in humans, *HRAS, KRAS,* and *NRAS,* encoding four RAS proteins (HRAS, KRAS 4A, KRAS 4B, and NRAS). These proteins are involved in the regulation of RAS – mitogen-activated protein kinase (RAS-MAPK) pathway, which regulates cell growth, proliferation, differentiation, and apoptosis. RAS proteins are central signal transduction molecules, which act as molecular switches through cycling between an active, GTP-bound, and an inactive, GDP-bound state. The first reports on mutations of *RAS* genes were from cancer studies and nowadays around 30% of all human cancers are known to have their origins in the mutation of one of the genes in the RAS-MAPK pathway, usually resulting in RAS hyperactivation (4).

**Figure 3:**
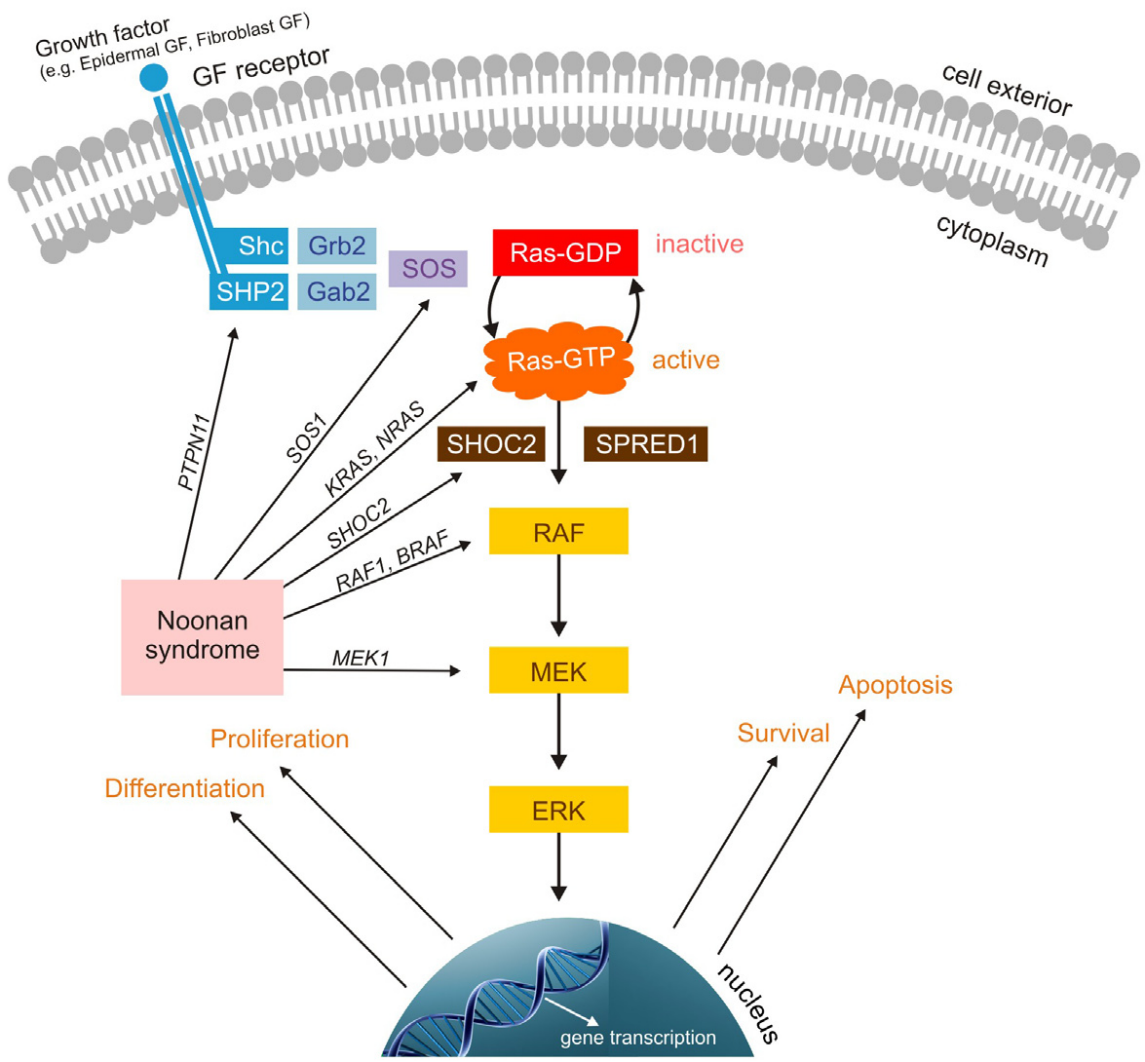
RAS-mitogen-activated protein kinase signaling pathway. Extracellular ligand such as growth factor (GF) binds to GF receptor and activates cytoplasmatic part of the receptor (a tyrosine kinase), which by phosphorylation enables binding with docking proteins such as GRB2. This protein forms a complex with sons of sevenless (SOS) guanine nucleotide exchange factor and activates it. Activated SOS removes guanosine diphosphate from RAS protein and activates it. Activated RAS protein then activates rapidly accelerated fibrosarcoma (RAF) kinase, and RAF kinase subsequently activates MEK kinase (mitogen-activated protein [MAP] kinase kinase). MEK kinase finally activates mitogen-activated protein kinase MAPK, also known as extracellular signal regulated kinase. Mutations in genes controlling production of these signaling proteins, causing Noonan syndrome, are indicated (*PTPN11, SOS1, KRAS, NRAS, SHOC2, RAF1, BRAF, MEK1*).

Furthermore, germline *KRAS* mutations are also found in developmental diseases like NS, and the overall contribution of *KRAS* mutations to this disease is around 5% (5). In addition to *KRAS* mutations, many other germline RAS-MAPK pathway mutations are also found in NS. Up to now, heterozygous germline mutations in seven different genes (*PTPN11, SOS1, RAF1, SHOC2, KRAS, BRAF, NRAS*) have been described. All these genes are encoding kinases and other proteins, and their mutations are connected with congenital heart disease, feeding difficulties, developmental delay, and short stature. These so called RASopathies include NS, cardio-facio-cutaneous syndrome, LEOPARD syndrome (lentigines, electrocardiographic anomalies, ocular hypertelorism, pulmonary stenosis, abnormal genitalia, and deafness), and Costello syndrome (Figure 3).

There are several genotype-phenotype correlations in NS, but no phenotypic feature is exclusively related to a specific genotype. HCM is present in about 20% of NS patients, but rapidly progressive HCM, resulting in an early death or a need for heart transplantation, occurs only sporadically in NS and other RASopathies. Described *KRAS* mutations (p.Lys5Asn, p.Val14Ile, p.Gln22Glu, p.Gln22Arg, p.Pro34Leu, p.Pro34Arg, p.Thr58Ile, p.Gly60Arg, p.Asp134Val, p.Phe156Leu, p.Val152Gly, and p.Asp153Val) are regularly associated with CHD (6-10). Extremely severe phenotype cases of NS and other RASopathies are typically caused only by specific mutations, resulting in overall signal flow dysregulation of the RAS-MAPK pathway (9,11,12).

We think that novel germline *KRAS* mutation due to heterozygous unclassified missense variant in exon 3: c.179G>T (p.Gly60Val) in our patient with clinical features of NS is very likely to be pathogenic, since other mutations of the same amino acid (p.Gly60Ser and p.Gly60Arg) are known to be pathogenic mutations in NS (6,13). The malignant clinical course of the disease in our patient and the fatal outcome in just a few months might therefore be connected to RAS-MAPK pathway hyperactivation and consequently to rapidly progressive HCM.

In conclusion, *KRAS* mutation is a known cause of NS with usually mild clinical course. There are some rare exceptions, when a specific mutation of this gene causes a severe phenotype with malignant clinical course, usually due to rapidly progressive HCM. The *KRAS* mutation c.179G>T (p.Gly60Val) identified in our patient might be associated with such lethal form of NS. Further biochemical and functional studies are needed to confirm this hypothesis.
